# Construction of T cell exhaustion model for predicting survival and immunotherapy effect of bladder cancer based on WGCNA

**DOI:** 10.3389/fonc.2023.1196802

**Published:** 2023-05-30

**Authors:** Yuwen Xue, Guanghui Zhao, Xiaoxin Pu, Fangdong Jiao

**Affiliations:** ^1^ Department of Pulmonary and Critical Care Medicine, Qilu Hospital of Shandong University, Jinan, China; ^2^ Peking University People’s Hospital, Qingdao Women and Children’s Hospital, Qingdao, China; ^3^ Medical Laboratory Center, Qilu Hospital (Qingdao) of Shandong University, Qingdao, China; ^4^ Department of Pulmonary and Critical Care Medicine, Qilu Hospital (Qingdao) of Shandong University, Qingdao, China; ^5^ Department of Urology Surgery, Qilu Hospital (Qingdao) of Shandong University, Qingdao, China

**Keywords:** bladder cancer, T cell exhaustion, WGCNA, survival, immunotherapy

## Abstract

**Introduction:**

The prognosis of bladder cancer (BLCA) and response to immune checkpoint inhibitors (ICIs) are determined by multiple factors. Existed biomarkers for predicting the effect of immunotherapy cannot accurately predict the response of BLCA patients to ICIs.

**Methods:**

To further accurately stratify patients’ response to ICIs and identify potential novel predictive biomarkers, we used the known T cell exhaustion (TEX)-related specific pathways, including tumor necrosis factor (TNF), interleukin (IL)-2, interferon (IFN)-g, and T- cell cytotoxicpathways, combined with weighted correlation network analysis (WGCNA) to analyze the characteristics of TEX in BLCA in detail, constructed a TEX model.

**Results:**

This model including 28 genes can robustly predict the survival of BLCA and immunotherapeutic efficacy. This model could divide BLCA into two groups, TEXhigh and TEXlow, with significantly different prognoses, clinical features, and reactivity to ICIs. The critical characteristic genes, such as potential biomarkers Charged Multivesicular Body Protein 4C (CHMP4C), SH2 Domain Containing 2A (SH2D2A), Prickle Planar Cell Polarity Protein 3 (PRICKLE3) and Zinc Finger Protein 165 (ZNF165) were verified in BLCA clinical samples by real-time quantitative chain reaction (qPCR) and immunohistochemistry (IHC).

**Discussion:**

Our findings show that the TEX model can serve as biological markers for predicting the response to ICIs, and the involving molecules in the TEX model might provide new potential targets for immunotherapy in BLCA.

## Introduction

Bladder cancer (BLCA) is one of the most common urological malignancies in the world, with nearly 430,000 people diagnosed with BLCA and approximately 165,000 deaths worldwide each year (1). BLCA is significantly more prevalent in men, accounting for 75% of new cases and deaths (2), and smoking is considered a high-risk factor for BLCA (3). BLCA is divided into non-muscle infiltrative (NMIBC) and muscle infiltrative (MIBC). Of these, about 70% are NMIBC (4), which is less invasive with a recurrence rate of 50-70% (5), and about 30% of NMIBC patients will progress to MIBC (6). Once newly diagnosed or progressed to MIBC, the tumor-specific 5-year survival rate decreased significantly even after undergoing radical cystectomy (RC) (7).

The high survival rate of NMIBC is attributed to the widespread use of transurethral resection of bladder tumors (TURBT) and the effectiveness of intravesical bacillus Calmette-Guérin (BCG) immunotherapy (8). Intravesical instillation of BCG is the first non-targeted immunotherapy approved by the FDA for the treatment of BLCA. Also, it makes BLCA one of the first cancers to utilize the concept of immunotherapy (9). This success may be attributed to urothelial BLCA being considered immunogenic and possessing the highest tumor mutational burden (TMB) of all cancers (10). Unfortunately, the initial response rate of BCG immunotherapy is high, but up to 42% of patients relapse after treatment, and 14% progress (11). In a recent study, Strandgaard et al. found that relapse after BCG treatment was caused by T cell exhaustion (TEX) (12). TEX is mainly manifested by the gradual loss of effector function of T cells, and the loss of memory T cell characteristics. But this exhaustion can be reversed, at least in part, mainly by blocking inhibitory pathways such as PD-1 (13).

Immune checkpoint inhibitors (ICIs) are a new generation of immunotherapeutic agents. In recent years, the use of ICIs has changed the treatment patterns for various cancers, including melanoma, kidney cancer, and lung cancer (2). These targeted immunotherapeutic agents, such as cytotoxic T cell lymphocyte associated protein-4 (CTLA-4) blocking antibody and programmed death pathway blocking antibody, has been extensively studied in localized or metastatic BLCA. Pembrolizumab and nivolumab targeting programmed cell death 1 (PD-1), and atezolizumab, avelumab and durvalumab targeting programmed death-ligand 1 (PD-L1) have been approved for the treatment of metastatic BLCA (14). In contrast, the overall response rate of ICIs in BLCA was relatively lower (15).

Theoretically, the immune surveillance function of the human immune system is responsible for identifying and removing tumor cells. However, the reasons that cancer cells escape from the onslaught of immune attack have not yet fully understood, eventually leading to tumor formation or recurrence (16). Loss of tumor cell antigens, lack of death receptor signaling, inadequate co-stimulatory signaling during T cell activation and/or enhancement of negative regulatory pathways, including increased inhibitory cytokines and high expression of immune checkpoint molecules, are all well-defined mechanisms for cancer cells to evade immune attack (17). ICIs work precisely by unlocking the inhibition of anti-cancer immunity caused by overexpression of immune checkpoints. Notably, studies have shown that targeting existed TEX markers, including PD-1/PD-L1 (18), CTLA-4 (19), T-cell immunoglobulin domain and mucin domain containing molecule-3 (TIM-3) (20), and T-cell immunoglobulin and ITIM domain (TIGIT) (21) pathways do not lead to complete recovery of T cell function, possibly due to the complex correlation of various immunosuppressive pathways. Therefore, identifying new immune checkpoints and/or simultaneous targeting of multiple immunosuppressive pathways is necessary.

Both cytotoxic CD4+ T cells and CD8+ T cells have been reported to be effector immune cells in BLCA and play an essential role in anti-tumor (22). However, it is well known that persistent antigen stimulation can cause TEX. A prominent feature of BLCA is a high degree of somatic mutation, which theoretically produces more tumor antigens (23), complicating the heterogeneity and exhaustion phenotype of T cells in BLCA. To address this issue, we comprehensively evaluated the enrichment of known specific pathways associated with TEX in BLCA samples in the TCGA database and constructed a new prognosis-related TEX model. Finally, the expression of critical characteristic genes and proteins were verified by real-time quantitative chain reaction (qPCR) and immunohistochemistry (IHC) in BLCA clinical samples.

## Materials and methods

### Clinical BLCA samples

Twenty-three pairs of frozen BLCA tissues and matched adjacent samples were obtained from Qilu Hospital (Qingdao) with all patients’ informed consent. All samples were used to detect the mRNA level of characteristic genes by qPCR, and six pairs of samples were used to detect the protein expression level of characteristic genes by IHC. This study was approved by the Ethics Committee of Qilu Hospital (Qingdao) of Shandong University. The clinical data of BLCA patients are shown in **Supplementary Table S1**.

### Data download and pre-processing

Normalized The Cancer Genome Atlas (TCGA)-BLCA gene expression data (TPM) and clinical data were downloaded from the UCSC Xena platform (http://xena.ucsc.edu/, accessed on 15 December 2022) and used as the training dataset for constructing the TEX model, which included 411 tumor samples and 19 paracancerous samples. The gene expression data (TPM) of normal bladder tissue in the GTEx database were also downloaded from the UCSC Xena platform, which included nine normal samples. The expression data and survival information of the GSE13507, GSE48276, and GSE19915 datasets were downloaded from the Gene Expression Omnibus (GEO) website (https://www.ncbi.nlm.nih.gov/geo/, accessed on 25 December 2022) as the validation datasets, where multiple probes corresponding to the same gene, the probe values were averaged.

Referring to the specific pathways related to TEX reported in previous studies (24), including tumor necrosis factor (TNF), interleukin (IL)-2, interferon (IFN)-γ, and T- cell cytotoxic pathways. The gene sets of the above pathways were downloaded from the MSigDB database (https://www.gsea-msigdb.org/gsea/msigdb, accessed on 15 December 2022).

### Screening candidate gene marker for building TEX signature

The single sample Gene Set Enrichment Analysis (ssGSEA) algorithm in the R package GSVA was used to calculate the enrichment scores of the gene sets of the above four pathways in the TCGA-BLCA samples. The R package survival performed univariate analysis to calculate the correlation between the enrichment scores and the prognosis of BLCA patients, including overall survival (OS) and progression-free interval (PFI). Wilcox test was used to calculate the correlation between the enrichment scores and the clinical features of BLCA patients, including age, gender, tumor grade, tumor stage, lymphatic invasion, and therapeutic effect. The mutation data of the genes in the above four pathways were downloaded from the TCGA database, and R package maftools drew the mutation map of the top 50 genes. The key modules related to the enrichment scores were screened using the weighted correlation network analysis (WGCNA). Genes in key modules were identified as candidate markers reflecting the TEX signature. Gene Ontology (GO) functional annotation and Kyoto Encyclopedia of Genes and Genomes (KEGG) enrichment analysis were performed on these candidate markers through the R package clusterProfiler.

### Construction and validation of prognosis-related TEX model

Based on the candidate genes screened above, markers whose expression was significantly associated with the prognosis of BLCA patients were further screened by univariate Cox regression analysis of R package survival and visualized by R package survminer. The prognosis-related markers were analyzed by lasso Cox regression using R package glmnet. The genes with non-zero regression coefficients were obtained as characteristic genes to construct a prognosis-related TEX model (Hereinafter referred to as the TEX model).

The risk score for each BLCA sample was calculated using the TEX model, the formula is A=∑*coef* gene *i* × *expr* gene *i*, where *coef* is the regression coefficient of the gene *i* in the model, and *expr* is the expression value of the gene *i*. The BLCA patients were divided into high- and low-risk groups based on the median risk score of all samples. The prognostic predictive efficacy of the constructed TEX model was analyzed in TCGA-BLCA datasets and external independent datasets. The R package maftools was used to visualize the gene mutations between the high- and low-risk groups. Differences in gene expression and clinical parameters between the two groups were also analyzed.

### Assessing the value of the TEX model as an independent prognostic predictor for BLCA patients

Based on the clinical features of BLCA patients in the TCGA-BLCA datasets, the Wilcox test of R package ggpubr was used to analyze the differences in the risk score among different groups, including age, gender, tumor grade, tumor stage, lymphatic invasion, etc. Univariate and multivariate Cox regression analyses of R package survival were used to observe whether the TEX model was an independent prognostic predictor of BLCA patients. In addition, a nomogram consisting of the TEX model with commonly used clinical features was constructed by R package rms to assess the model’s efficacy in predicting the progression of BLCA patients.

### Association analysis of the TEX model and drug treatment effect

Based on the Genomics of Drug Sensitivity in Cancer (GDSC) database (https://www.cancerrxgene.org/, accessed on 20 January 2023), the Wilcox test of R package ggpubr was used to analyze the difference in chemotherapeutic drug sensitivity between high- and low-risk groups, and the differential expression of characteristic genes in the TEX model. R package Corrplot was used to analyze the correlation between the expression of characteristic genes and chemotherapeutic drug sensitivity.

The TEX model’s efficacy in predicting immunotherapy’s effect in BLCA patients was evaluated by three visualization forms: KM curves drawn by R package survival, scatter plots drawn by R package ggpubr, and ROC curves drawn by R package pROC. Referring to previous reports (25), the TIP tool (http://biocc.hrbmu.edu.cn/TIP/, accessed on 30 January 2023) was used to analyze the anti-cancer immune process activity between the high- and low-risk groups of TCGA-BLCA patients, and the Wilcox test of R package ggpubr analyzed the difference.

### qPCR

Total RNA was extracted from the tumor and matched adjacent tissue samples using TRIzol LS reagent (Thermo Fisher Scientific, USA). The cDNA was synthesized by Reverse Transcriptase M-MLV (Takara, Japan) following the manufacturer’s instructions. In addition, qRT-PCR was performed with SYBR^®^ Premix Ex Taq™ II (Takara, Japan). GAPDH was used as an endogenous control for qPCR. All primers of twelve characteristic genes of the TEX model were listed in **Supplementary Table S2**. The relative expression was compared using 2−^ΔCt^ between tumor and adjacent samples, with ΔCt = Ct_characteristic genes_ – Ct_GAPDH._

### IHC

For IHC, 5-micron frozen sections were fixed in pre-cooled acetone. After washing and antigen retrieval, sections were placed in 3% hydrogen peroxide to block endogenous peroxidase. After antigen blocking, primary and secondary antibodies were added for incubation. The specific information on the antibodies used is shown in **Table S3**. After DAB color development and cell nucleus restraining, dehydration and mounting were performed. Protein expression levels were analyzed by Image-Pro Plus software (Media Cybernetics, USA).

## Results

### Correlation of specific TEX-related pathways with prognosis and clinical features of BLCA patients

**Figure 1:**
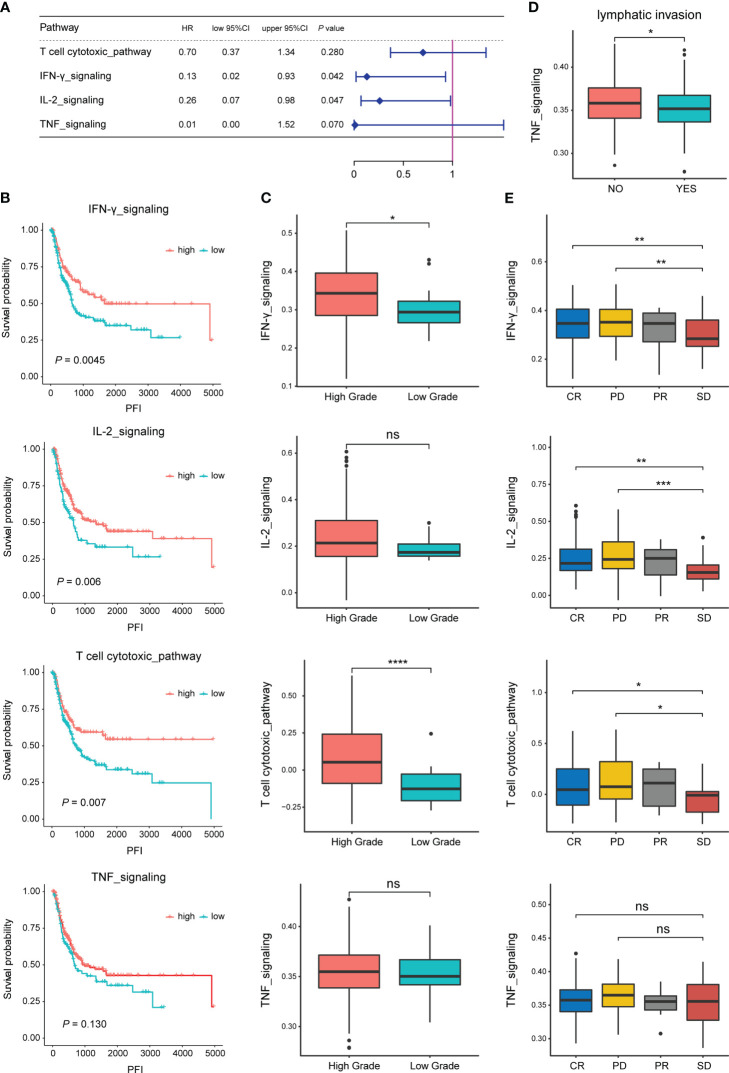
Correlation of specific pathways related to TEX with the prognosis and clinical features of BLCA patients. **(A)** Correlation analysis of the enrichment scores of four pathways and the OS of BLCA patients in the TCGA dataset. **(B)** Survival analysis (PFI) of BLCA patients in high and low enrichment score groups of the four pathways. **(C)** Difference analysis of enrichment scores of the four pathways between high and low tumor grade groups. **(D)** Difference analysis of TNF pathway enrichment score between groups with or without lymph node metastasis. **(E)** Difference analysis of enrichment scores of the four pathways among different treatment effect groups. *p-value < 0.05, **p-value < 0.01, ***p-value < 0.001, ns, not significant.

Analysis of the correlation between the enrichment scores of four specific pathways related to TEX in each TCGA-BLCA sample and patients’OS revealed that the high enrichment of IL-2 signaling and IFN-γ signaling was significantly positively correlated with the OS of BLCA patients (*P*<0.05, **Figure 1A**). BLCA patients were classified into high and low groups based on the enrichment of each pathway using the optimal threshold. The results showed that the enrichment scores of T-cytotoxic pathway, IFN-γ signaling, and IL-2 signaling significantly affected the PFI of BLCA patients. Similarly, the patients with lower enrichment scores had shorter PFI time (*P*<0.01, **Figure 1B**). Interestingly, the difference analysis of the enrichment scores of the four pathways among different clinical features showed that IFN-γ signaling and T-cytotoxic pathway were significantly highly enriched in samples with higher tumor grades (*P*<0.05), the other two pathways also showed this trend (**Figure 1C**), suggesting that the specific pathways related to TEX may become more abnormal in patients with a higher malignant degree. In addition, the enrichment score of TNF signaling was negatively correlated with lymph node metastasis in BLCA patients (*P*<0.05, **Figure 1D**). There was also a specific correlation between the enrichment scores of the four pathways and the therapeutic effect. The enrichment scores of patients with complete response (CR) and progressive disease (PD) were significantly higher than those of patients with stable disease (SD), suggesting that the activity of the pathways involved has an important impact on the development of the disease. There may be more significant heterogeneity among BLCA patients (**Figure 1E**).

### Candidate markers for building TEX signature

**Figure 2:**
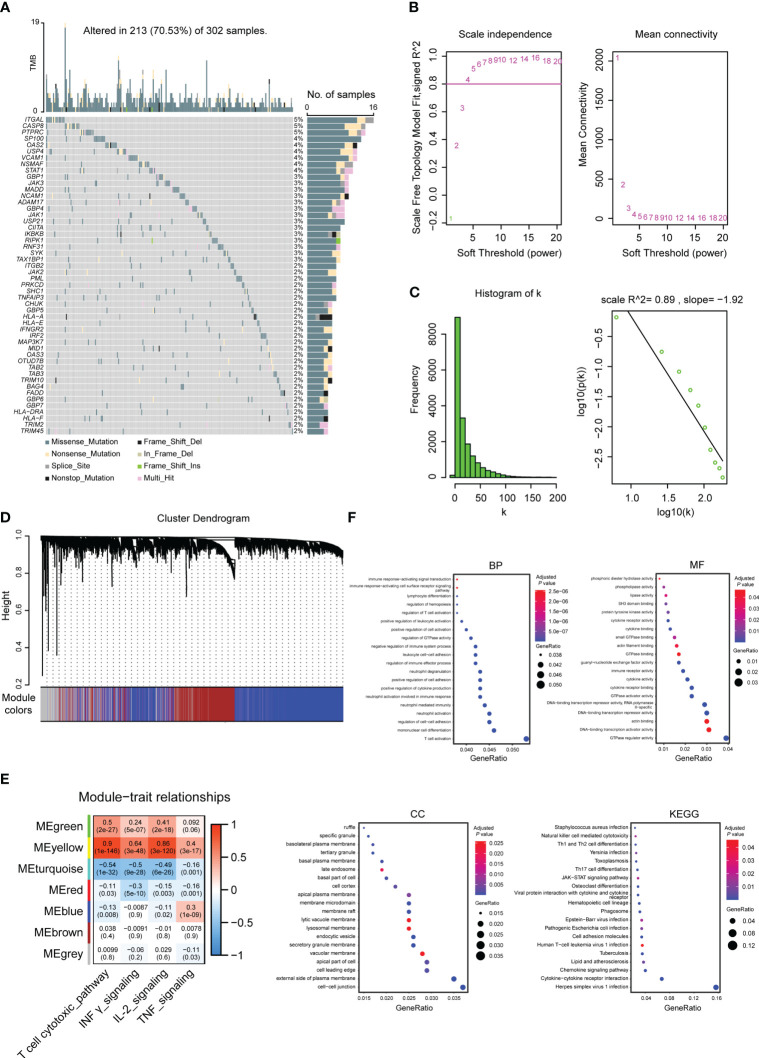
Molecular characterization and functional enrichment analysis of genes used to build TEX signatures. **(A)** Top 50 gene mutation maps in specific pathways related to TEX. **(B)** Soft-threshold screening and scale-free fit index plots. **(C)** Scale-free topological graph when b is equal to 5. **(D)** Hierarchical clustering dendrogram. **(E)** Module-phenotype correlation heatmap. **(F)** GO (BP, MF, CC) and KEGG enrichment analysis of marker genes used to build TEX signatures, the bluer the color was, the weaker the difference was, while the red was the opposite.

The gene set of the four pathways includes a total of 160 genes, of which 147 genes have obtained mutation data through the TCGA database. The mutation map of the top 50 genes with the highest mutation rate is shown in **Figure 2A**. The results showed that missense mutations dominated the mutation type.

Key modules associated with enrichment scores were screened using WGCNA analysis. According to the soft threshold and the scale-free fitting index, it was found that there was no intersection point between power and the horizontal line (correlation 0.8) (**Figure 2B**). Referring to the previously reported method (26), the estimated value of powerEstimate equal to 5 is selected as the β value. It can be seen that k is negatively correlated with p(k) (correlation coefficient 0.89>0.8) (**Figure 2C**), indicating that the selected β value is suitable for the establishment of a gene scale-free network. Six modules were obtained (**Figure 2D**), and two modules, Meyellow with a significant positive correlation and Meturquoise with a significant negative correlation, were selected according to the correlation between modules and enrichment scores. A total of 4571 genes were included as candidate markers for TEX (**Figure 2E**). The R package clusterProfiler was used to perform GO and KEGG analysis on the obtained 4571 genes, and the top 20 pathways were selected for display (**Figure 2F**).

### Prognosis-related TEX model

**Figure 3:**
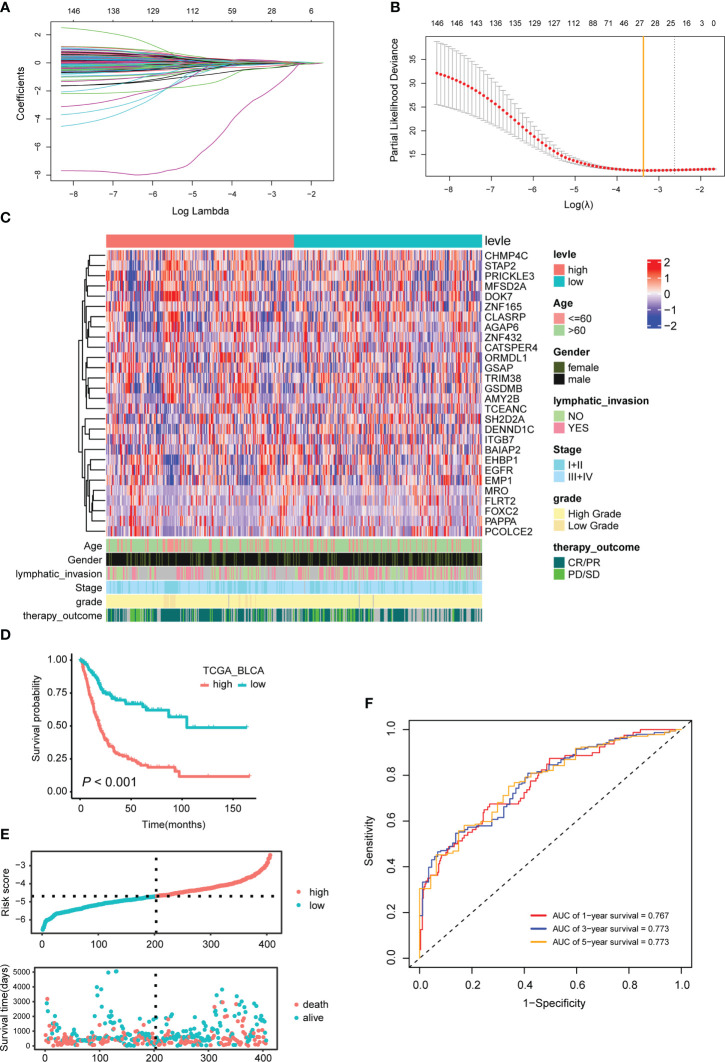
Characteristic genes used to construct the TEX model and their effects on the prognosis of BLCA patients. **(A)** LASSO coefficient profiles of the 147 prognosis-related TEX markers. **(B)** Twenty-eight characteristic genes were selected using LASSO Cox regression analysis. **(C)** Expression heatmap of 28 signature genes. **(D)** The KM survival curve for TCGA_BLCA samples with high- and low-risk scores is based on the TEX model. **(E)** The risk score and survival time distribution. The upper figure represents the risk score of each patient, and the lower figure corresponds to the patient’s survival status and survival time. **(F)** Time-dependent ROC curves for 1, 3 and 5 years.

Based on the above 4571 candidate marker for TEX, univariate Cox was used to screen OS-related genes, and a total of 147 genes were found with *P*<0.001 as a threshold. Based on the 147 prognostic-related TEX markers, according to the previous method (27), 28 characteristic genes with non-zero coefficients were obtained for constructing the prognosis-related TEX model using LASSO Cox analysis of R package glmnet (**Figures 3A–C**).

### The TEX model is an independent prognostic predictor for BLCA patients

Based on the median risk score of the TEX model, the TCGA-BLCA samples were divided into two groups, namely TEX^high^ (n=201) and TEX^low^ (n=202) (**Table S4**). Survival analysis showed that BLCA patients in the TEX^high^ group had a shorter OS (*P*<0.001, **Figures 3D, E**). The ROC curve results showed that the TEX model’s AUCs for predicting patients’ survival at 1, 3, and 5 years were 0.767, 0.773, and 0.773, respectively, which were all greater than 0.75. The TEX model has high accuracy in predicting the prognosis of BLCA patients (**Figure 3F**).

The effect of the TEX model on the prognosis of BLCA patients was validated in multiple independent external datasets. Based on the best cut-off values of TEX model risk scores for all samples, BLCA patients in GSE13507 dataset were divided into TEX^high^ and TEX^low^ groups. The results showed that patients in the TEX^high^ group had shorter OS (*P* = 0.026, **Figure S1A**). Similar results were obtained in the GSE48276 and GSE19915 datasets (*P* = 0.049 and *P* = 0.012, **Figures S1B, C**).

**Figure 4:**
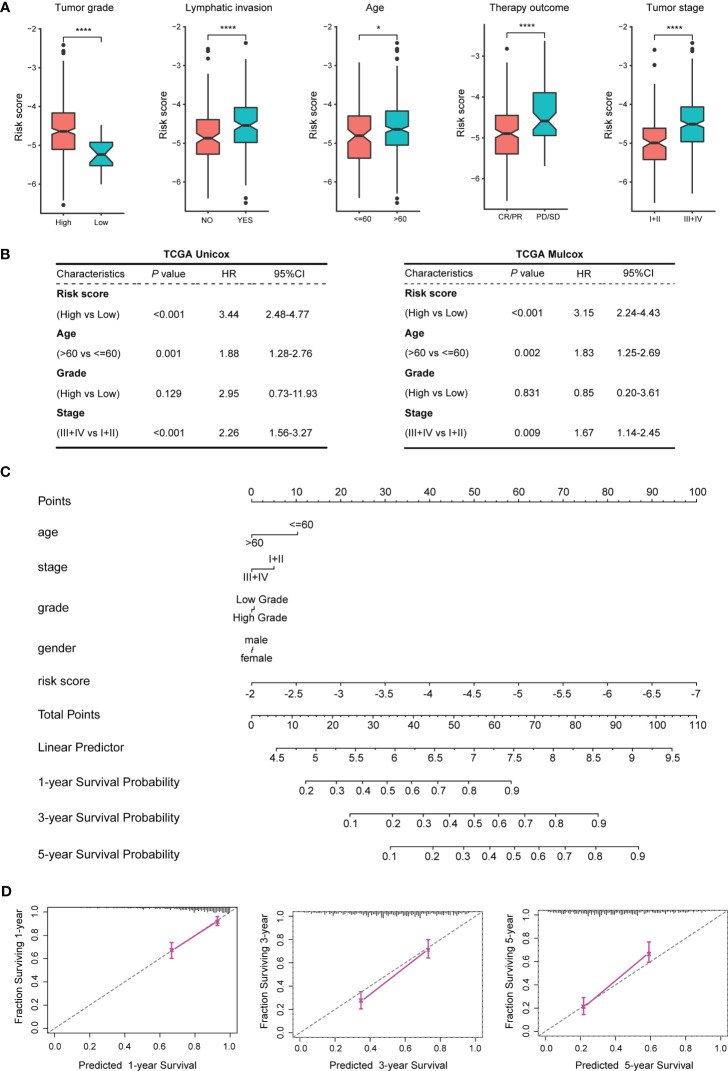
Correlation analysis between the TEX model and clinical features of BLCA patients. **(A)** Correlation analysis between the TEX model and various clinical features. **(B)** Univariate and multivariate Cox regression analysis of risk scores and clinical features of TCGA_BLCA samples. **(C)** The nomogram constructed by the TEX model combined with several clinical features. **(D)** 1, 3, 5-year survival probability prediction map. *p-value < 0.05, ****p-value < 0.0001.

The patients in the training dataset TCGA_BLCA were stratified according to different clinical characteristics. The results showed that the risk scores of the TEX model were closely related to age, tumor stage, tumor grade, lymph node metastasis, and treatment effect. BLCA patients with older age, higher tumor stage or grade, positive lymph nodes, and SD or PD after treatment had higher risk scores (**Figures 4A**; **S2**).

Univariate and multivariate Cox regressions were used to analyze the prognostic correlation between the TEX model and the clinical features of the training dataset TCGA_BLCA. The results of both univariate analysis (*P* < 0.001, HR =3.44, 95%CI = 2.48-4.77) and multivariate analysis (*P* < 0.001, HR=3.15, 95%CI = 2.24-4.43) indicated that TEX model could be used as an independent risk factor for BLCA patients (**Figure 4B**). The univariate and multivariate Cox regression analysis in the external validation dataset GSE13507 also showed that the TEX model could be an independent risk factor for BLCA patients (**Figure S3**). Using the TEX model and multiple clinical parameters, including age, stage, grade, gender, etc., to construct a nomogram to predict the prognosis of BLCA patients in 1, 3, and 5 years (**Figure 4C**). Survival probability prediction plots indicate that the constructed nomogram exhibited a strong prognostic predictive ability (**Figure 4D**).

### The TEX model is closely related to the sensitivity of many chemotherapeutic drugs

**Figure 5:**
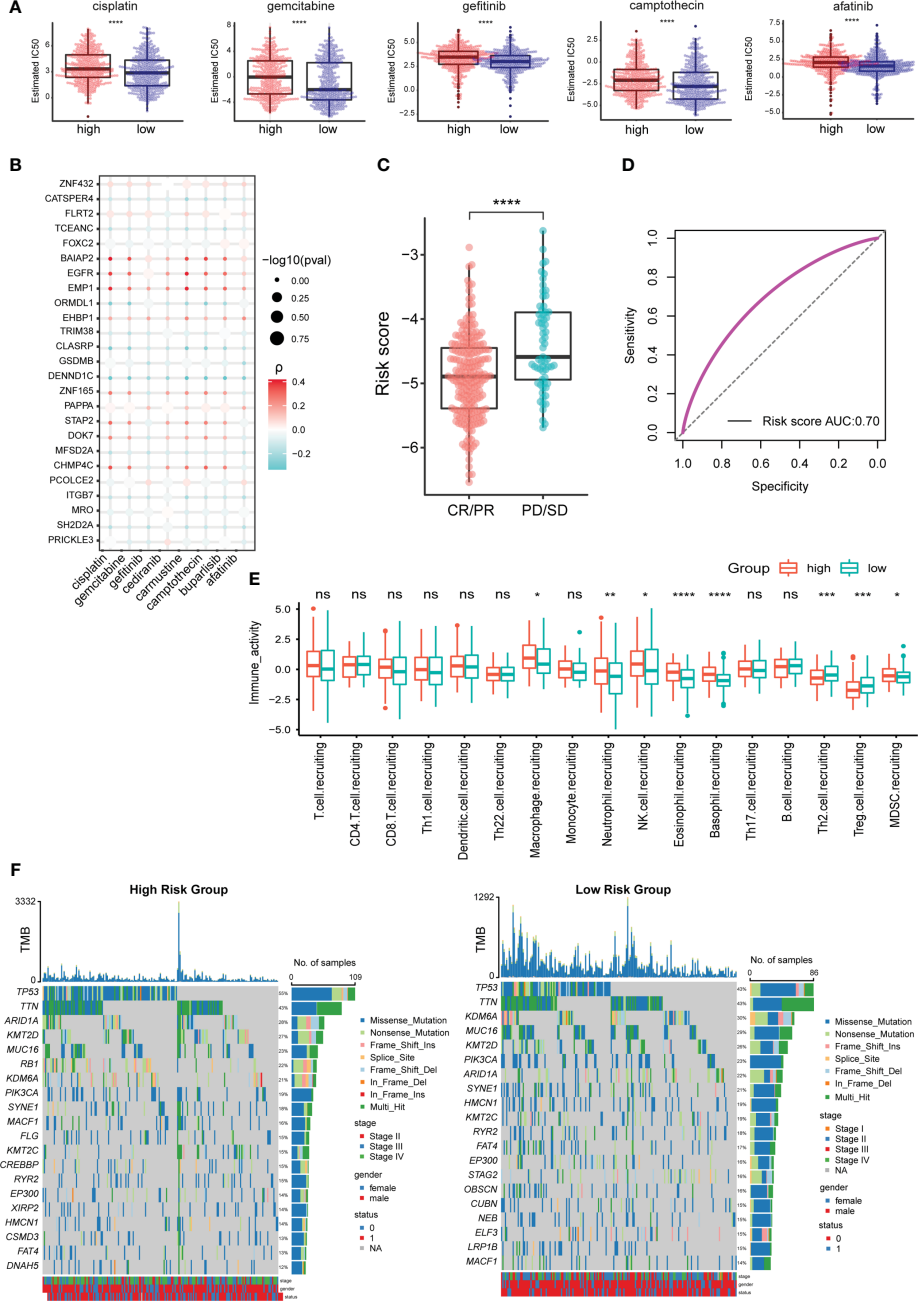
TEX model is related to the effect of chemotherapy and immunotherapy in BLCA patients. **(A)** Difference analysis of estimated IC50 values of chemotherapy drugs among TCGA_BLCA samples with high- and low-risk scores. **(B)** Correlation analysis between expression levels of characteristic genes and estimated IC50 values of chemotherapeutic drugs. **(C)** Difference analysis of risk scores between different immunotherapy effect groups. **(D)** ROC curves for predicting the effect of immunotherapy based on the TEX model. **(E)** Differential analysis of anti-cancer immuneprocess activity between TCGA_BLCA samples with high- and low-risk scores. **(F)** The 20 most frequently mutated genes in the high- and low-risk groups. *p-value < 0.05, **p-value < 0.01, ***p-value < 0.001, ****p-value < 0.0001, ns, not significant.

Wilcox test was used to analyze the difference in sensitivity of chemotherapeutic drugs between the TEX^high^ and TEX^low^ groups. The results showed that cisplatin, gemcitabine, gefitinib, camptothecin, and afatinib had higher estimated IC50 values in the TEX^high^ group (**Figure 5A**). When the 25 characteristic genes used to construct the TEX model were divided into high- and low-expression groups according to their median expression values, it was found that the expression of 21 genes significantly impacted the sensitivity of chemotherapy drugs (**Figure S4**). The correlation analysis between the expression levels of 25 characteristic genes and the sensitivity of chemotherapeutic drugs intuitively showed that the high expression of epidermal growth factor receptor (EGFR), epithelial membrane protein 1 (EMP1), and EH domain binding protein 1 (EHBP1) was positively correlated with higher IC50 value. In contrast, the increased expression of transcription elongation factor A N-Terminal and central domain containing (TCEANC), gasdermin B (GSDMB), cation channel sperm associated 4 (CATSPER4) and CLK4 associating serine/arginine rich protein (CLASRP) was related to the lower IC50 value (**Figure 5B**).

### The TEX model can predict immunotherapy response in BLCA patients

Immunotherapy is one of the important methods for the clinical treatment of BLCA patients. The analysis results of the prognosis-related data of BLCA patients receiving immunotherapy showed that the SD/PD group had a higher risk score than the CR/PR group (**Figure 5C**). The AUC value of the ROC curve was 0.7 (**Figure 5D**), so the TEX model can better predict the effect of immunotherapy in BLCA patients. The difference analysis of anti-cancer immune process activity between high- and low-risk groups showed that the recruitment process of CD8+ T cells (no significant difference), macrophages, neutrophils, NK cells, and Myeloid-derived suppressor cells (MDSCs) were more active in TEX^high^ group. In contrast, Th2 and regulatory T (Treg) cells were more involved in the TEX^low^ group (**Figure 5E**).

We analyzed the 20 most frequently mutated genes in the high- and low-risk groups to explore the underlying molecular mutations behind the differences in immune activity processes. The results showed that the mutation rate of tumor suppressor gene was higher in the TEX^high^ group, such as tumor protein P53 (TP53) (55% vs 43%), AT-rich interaction domain 1A (ARID1A) (28% vs 22%) and lysine methyltransferase 2D (KMT2D) (27% vs 26%). It is particularly striking that the mutation rate of RB transcriptional corepressor 1 (RB1) was as high as 22% in the TEX^high^ group while less than 14% in the TEX^low^ group (**Figure 5F**). RB1 is an immune-related prognostic biomarker and a promising target for immunotherapy in several cancers, including BLCA (28, 29).

### Verification of characteristic gene expression in clinical BLCA samples

**Figure 6:**
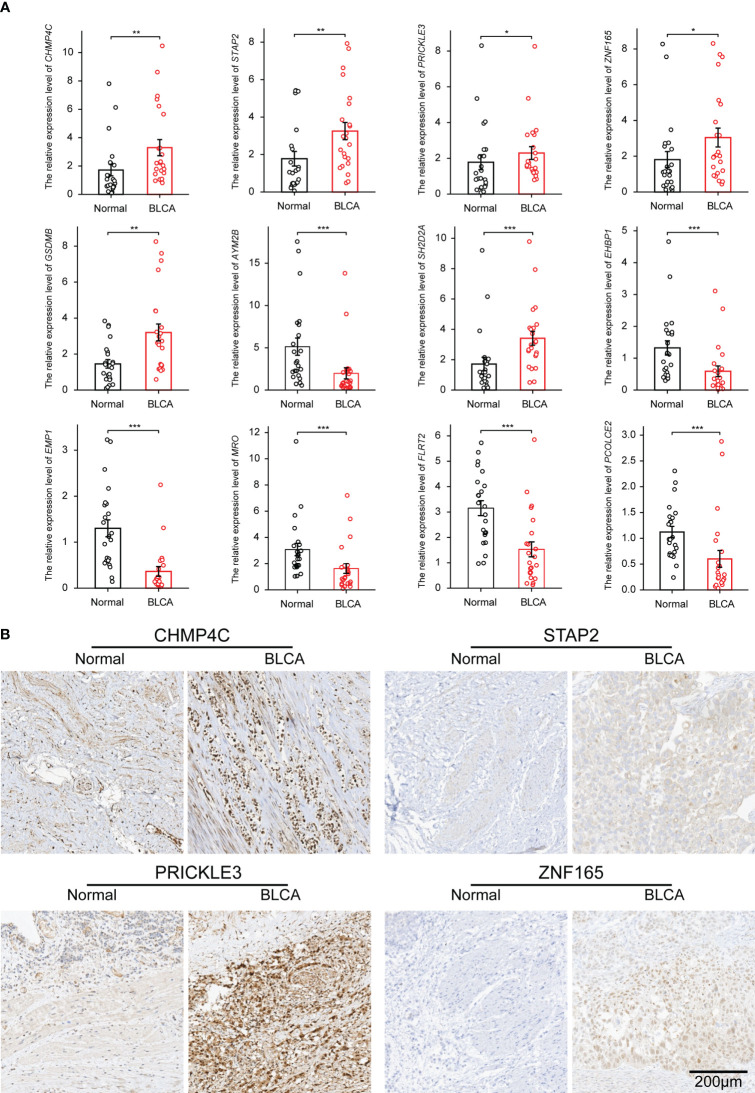
The expression of characteristic genes was verified in clinical BLCA samples by qPCR and IHC. **(A)** The differential expression of mRNA of 12 characteristic genes in 23 pairs of BLCA samples. **(B)** IHC results of 4 genes highly expressed in BLCA tissues. *p-value < 0.05, **p-value < 0.01, ***p-value < 0.001.

In view of the importance of the 28 characteristic genes in model construction, we analyzed their expression differences between the TEX^high^ and TEX^low^ groups, and the results showed that nine genes were highly expressed in the TEX^high^ group, while 19 were highly expressed in the TEX^low^ group (**Figure S5**). To facilitate the use of the TEX model in clinical practice, we alternatively performed the differential expression analysis of 28 characteristic genes in TCGA and GTEx datasets, including 407 BLCA samples and 28 paracancerous or normal bladder tissue samples. The results showed that Charged Multivesicular Body Protein 4C (CHMP4C), Signal Transducing Adaptor Family Member 2 (STAP2), Prickle Planar Cell Polarity Protein 3 (PRICKLE3), Zinc Finger Protein 165 (ZNF165), Gasdermin B (GSDMB) and SH2 Domain Containing 2A (SH2D2A) were highly expressed in bladder cancer. In contrast, Amylase Alpha 2B (AMY2B), EH Domain Binding Protein 1 (EHBP1), Epithelial Membrane Protein 1 (EMP1), Maestro (MRO), Fibronectin Leucine Rich Transmembrane Protein 2 (FLRT2) and Procollagen C-Endopeptidase Enhancer 2 (PCOLCE2) were low expressed (**Figure S6A**). We then performed differential expression analysis on 19 paired samples (BLCA tissues and paired paracancerous tissues) in the TCGA dataset. The results showed a similar expression trend (**Figure S6B**). To further verify the results, we detected the above 12 characteristic genes by qPCR in 23 pairs of BLCA and paracarcinoma samples, and the results were consistent with the database analysis (**Figure 6A**). Six characteristic genes highly expressed in BLCA comparing to paracarcinoma were verified by IHC (**Figures 6B**; **S7**).

## Discussion

Anti-tumor immunity and immune escape are always in a dynamic game in the tumor formation and development process. CD8+ T cells usually differentiate into a functionally exhausted phenotype after continuous exposure to tumor antigens, but this is a dynamic and partially reversible process (30). This process encompasses a wide range of phenotypic switch and continuous intermediate states, which makes it challenging to use a few markers to fully and accurately define the exhaustion level of the functional state of T cells. TEX involves high expression of multiple inhibitory receptors, such as PD1, PD-L1, TIM-3, TIGIT, CTLA-4, and altered usage of transcription factors, such as transcription factors T cell factor (TCF1), thymocyte selectionassociated HMG BOX (TOX) and T-bet (31). It also included hierarchical dysfunction of cellular effectors, i.e., the production of IFN- γ, TNF, and IL-2 (32). Zhang et al. established a compendium of TEX-specific pathways, including TNF, IL-2, IFN-γ, and T- cell cytotoxic pathways, and proposed a TEX-based immunotyping scheme through pan-cancer analysis (24). Based on this study, we combined WGCNA analysis to analyze the TEX features in BLCA.

We found that the high enrichment scores of IL-2 and INF- γ pathways were positively correlated with the survival of BLCA patients, including OS and PFI. The discovery of IL-2 is an essential milestone in immunotherapy, which increases the production of cytotoxic lymphocytes and activates NK cells and B cells (33). Interferon, as a glycoprotein, also plays an important role in anti-tumor cell proliferation and immune regulation (34). Applying IL-2 and IFN in BLCA has achieved initial results (35, 36). The enrichment of these TEX-specific pathways also has a complex correlation with tumor grade, lymph node metastasis, and therapeutic efficacy, suggesting that the existed TEX features still have considerable heterogeneity in BC patients.

The activation state of TEX-specific pathways is closely related to the mutation of key signal molecules. ITGAL, also known as CD11A, has the highest mutation rate in 147 TEX-specific pathway-related genes and belongs to the T- cell cytotoxic pathway gene set. Its gene polymorphisms have been proven to impact the human immune response, including allergic diseases (37). In the aspect of tumors, ITGAL is an independent prognostic biomarker of myeloid leukemia and gastric cancer, and its mediation of tumor occurrence and progression is closely related to the regulation of immune function (38, 39). The functional role of ITGAL in bladder cancer has not been focused on and is a potential research direction. Our analysis of BLCA samples in the combined TCGA and GTEx databases revealed that ITGAL was significantly low expressed in bladder cancer tissues (data not shown), suggesting that ITGAL may have a different regulatory mechanism in bladder tumors.

We constructed a prognostic-associated TEX model consisting of 28 genes, which has strong predictive power for the prognosis of BLCA patients and response to chemotherapy and immunotherapy. Twelve genes with significantly altered expression in BLCA were verified by qPCR and IHC, among which CHMP4C, STAP2, PRICKLE3, ZNF165, GSDMB, and SH2D2A were significantly highly expressed in BLCA. Interestingly, most of these genes are related to the infiltration of immune cells and/or the formation of TIME. For example, CHMP4C, a protein involved in membrane remodeling, has been frequently spotlighted in several recent reports. Its high expression has been shown to promote the occurrence and progression of various tumors, including lung cancer, pancreatic cancer, hepatocellular carcinoma, and cervical cancer (40, 41). Wu et al. constructed a risk model based on CHMP4C to predict the tumor immune microenvironment (TIME) and guide the chemotherapy and immunotherapy of BLCA (42). ZNF165 is a functionally enigmatic cancer/testis (CT) antigen whose expression is usually restricted to germ cells but is aberrantly activated in tumors and is associated with the prognosis of a variety of tumors, mediated mainly by participating in the regulation of the TIME (43, 44). STAP2 promotes the generation of long-term functional memory CD8+ T cells by preventing terminal effector differentiation (45). Li et al. constructed an immune-related risk characteristic based on eight genes, including STAP2, which can predict the anti-PD-L1 efficacy of urothelial cancer (46). The role and function of GSDMB in the tumor are being widely studied, and its increased expression is related to immune infiltration and poor prognosis (47). The SH2D2A gene encoding for a T cell-specific adapter protein controls early T cell activation (48).

These signature genes are novel TEX biomarkers and potential immunotherapy targets. There are some limitations to this study. Firstly, the TEX-specific pathways we selected can not fully define the molecular characteristics of TEX, although we used WGCNA analysis to provide a targeted characterization of TEX in BLCA. Secondly, the immune cells that exert anti-tumor effects in the tumor immune microenvironment include T cells, NK cells, etc. The complex immunosuppressive regulation mechanism makes it difficult to restore the function of T cells fully. Third, the TEX model we constructed needs to be validated in more clinical patients, including TEX^high^ and TEX^low^ grouping study. Fourth, the number of genes involved in the construction of the model is large, which increases the difficulty and complexity of its application in clinical practice. We will further study and optimize this model.

## Data availability statement

The raw data supporting the conclusions of this article will be made available by the authors, without undue reservation.

## Ethics statement

The studies involving human participants were reviewed and approved by Ethics Committee of Qilu Hospital (Qingdao) of Shandong University. The patients/participants provided their written informed consent to participate in this study.

## Author contributions

YX and GZ designed the paper and performed bioinformatics analysis. XP completed the data collection from the database. GZ and FJ completed the data verification and partial information analysis. FJ critically reviewed and edited the manuscript. All authors contributed to the article and approved the submitted version.
