# Supplementary material for: Construction of T cell exhaustion model for predicting survival and immunotherapy effect of bladder cancer based on WGCNA
Source: Front Oncol. 2023 May 30;13:1196802. doi: 10.3389/fonc.2023.1196802 (PMC10266200; doi:10.3389/fonc.2023.1196802)
Supplement: Supplementary file 1 [file DataSheet_1.docx]

**Figure Legends**

**Figure S1** The KM survival curve and time-dependent ROC curves for BLCA samples in GSE13507 (A), GSE48276(B), and GSE19915 datasets (C) with high- and low-risk scores based on the TEX model.

**Figure S2** (A-H) Comparison of clinical parameters between TEX^high^ and TEX^low^ groups.

**Figure S3** Univariate and multivariate cox regression analysis of risk scores and clinical features of BLCA samples in the GSE13507 dataset.

**Figure S4** Difference analysis of estimated IC50 values of chemotherapy drugs between high and low expression groups of 21 characteristic genes.

**Figure S5** Differential expression of 28 characteristic genes between TEX^high^ and TEX^low^ groups.

**Figure S6** Differential expression analysis of 28 characteristic genes in the TCGA and GTEx datasets. (A) Differential expression of 28 characteristic genes in 407 BLCA and 28 control samples. (B) Differential expression of 28 characteristic genes in 19 paired BLCA samples.

**Figure S7** IHC results of the remaining two genes highly expressed in BLCA tissues.
