# Supplementary figures and images for: Construction of T cell exhaustion model for predicting survival and immunotherapy effect of bladder cancer based on WGCNA
Source: Front Oncol. 2023 May 30;13:1196802. doi: 10.3389/fonc.2023.1196802 (PMC10266200; doi:10.3389/fonc.2023.1196802)

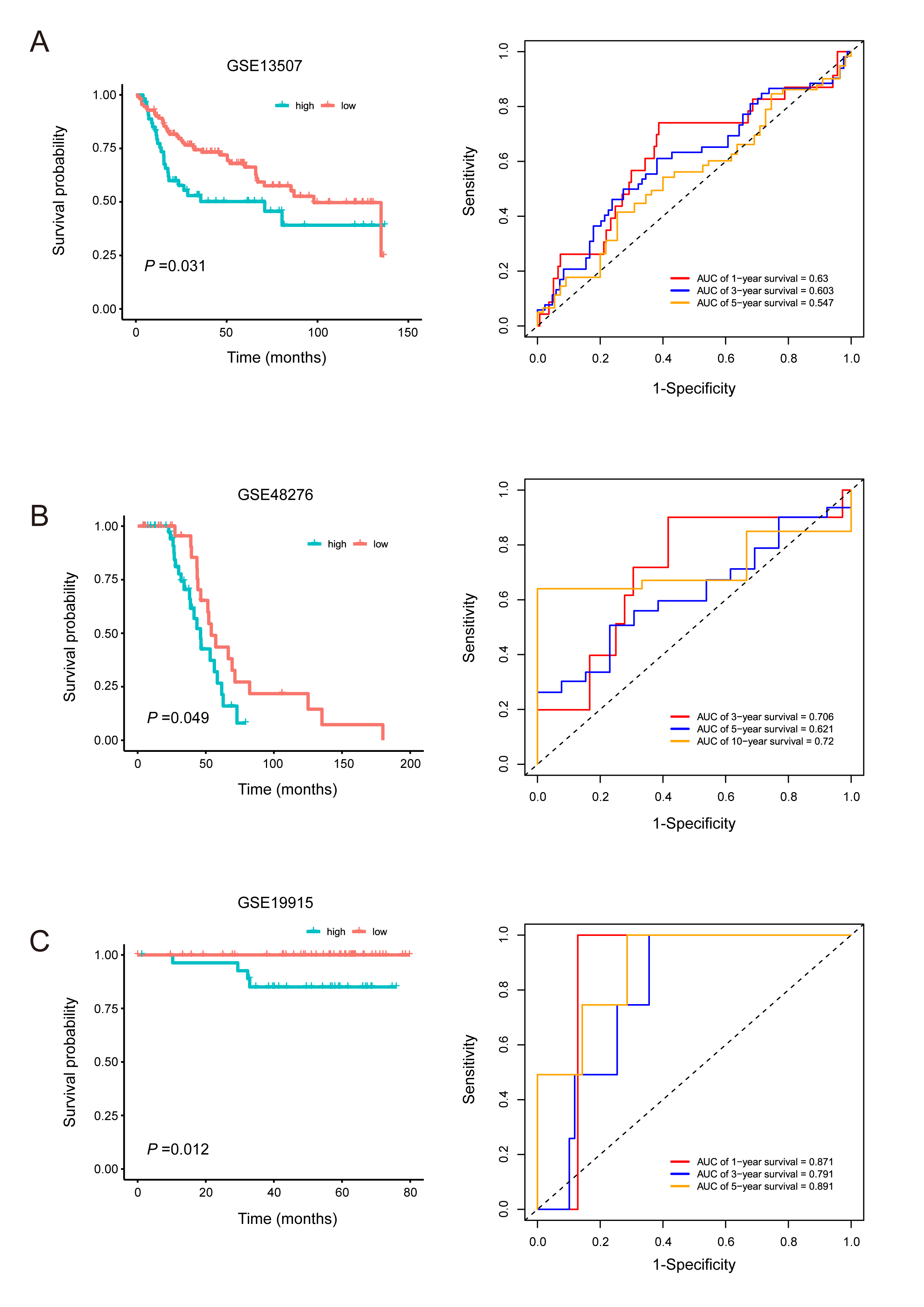

Supplement: Supplementary file 2 [file Image_1.tif]

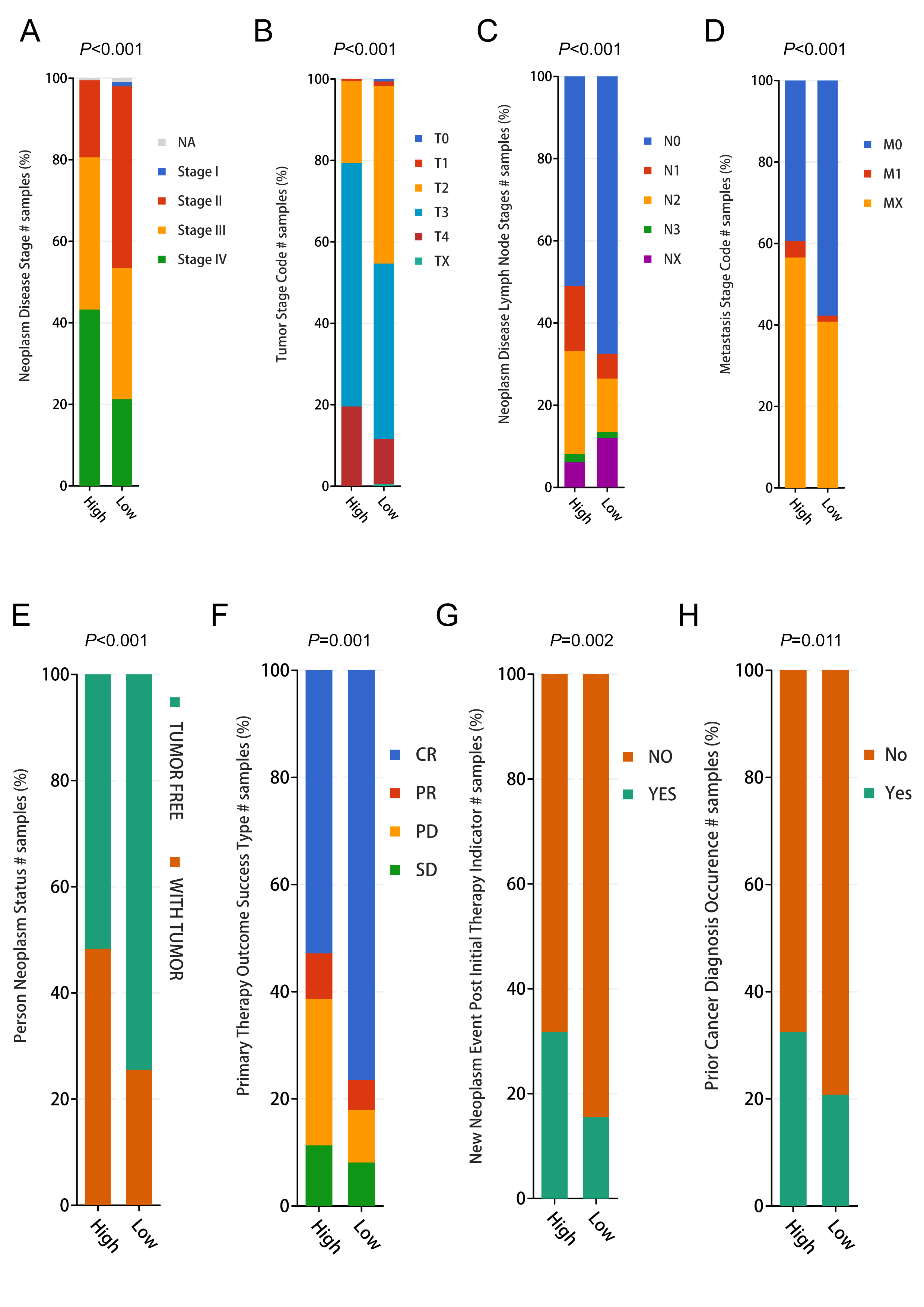

Supplement: Supplementary file 3 [file Image_2.tif]

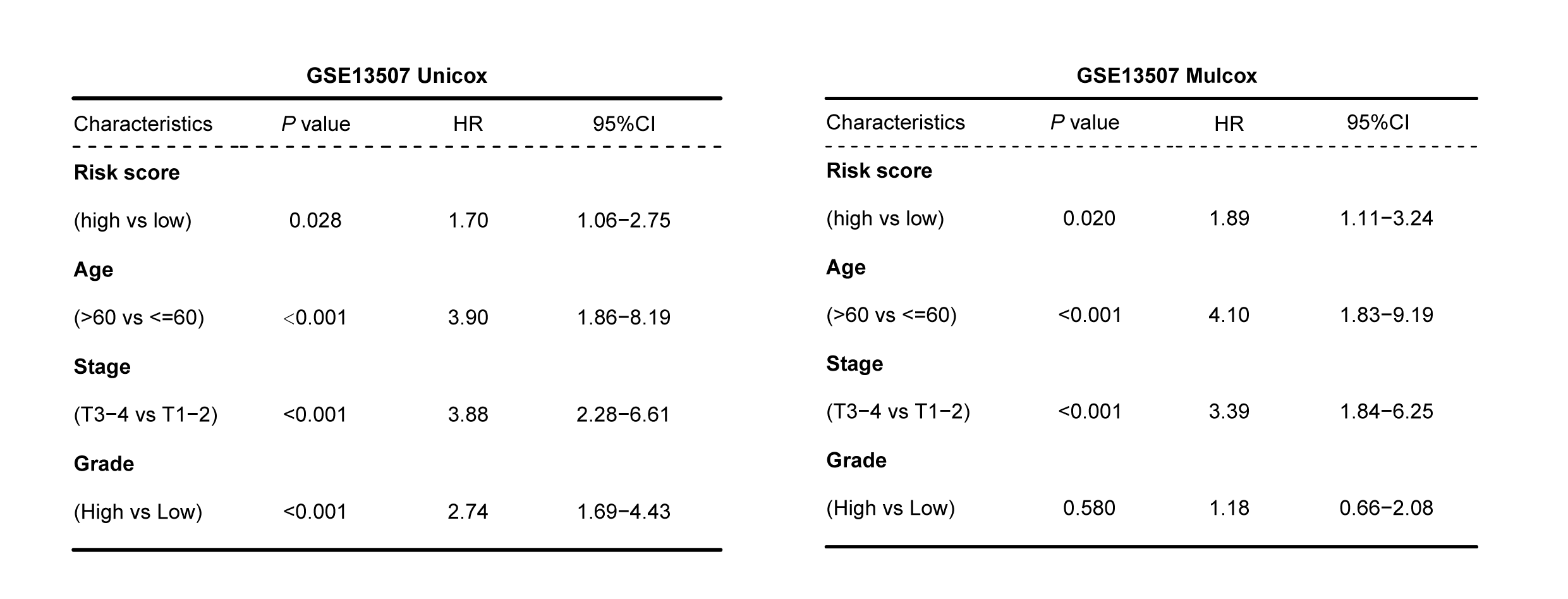

Supplement: Supplementary file 4 [file Image_3.tif]

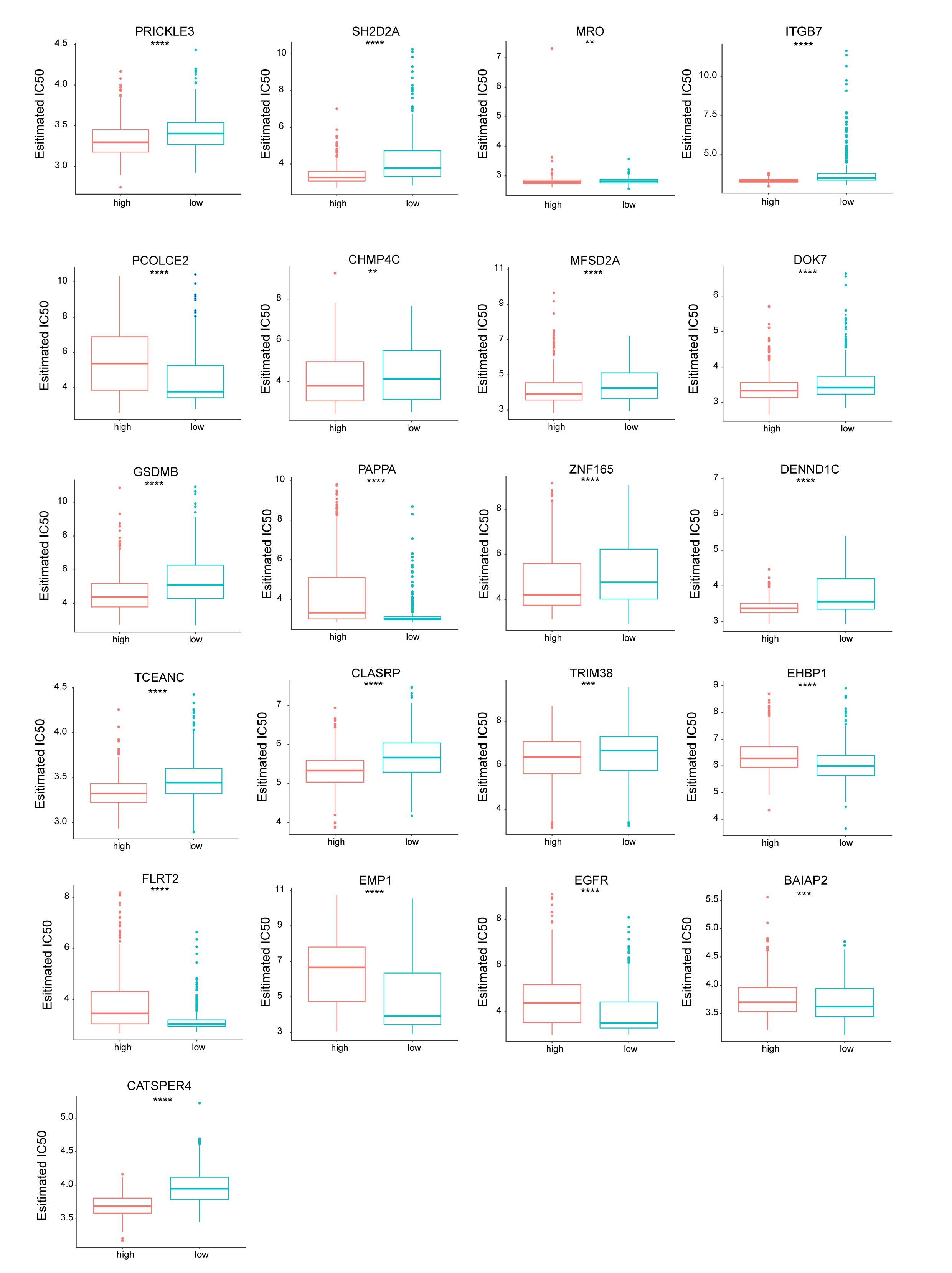

Supplement: Supplementary file 5 [file Image_4.tif]

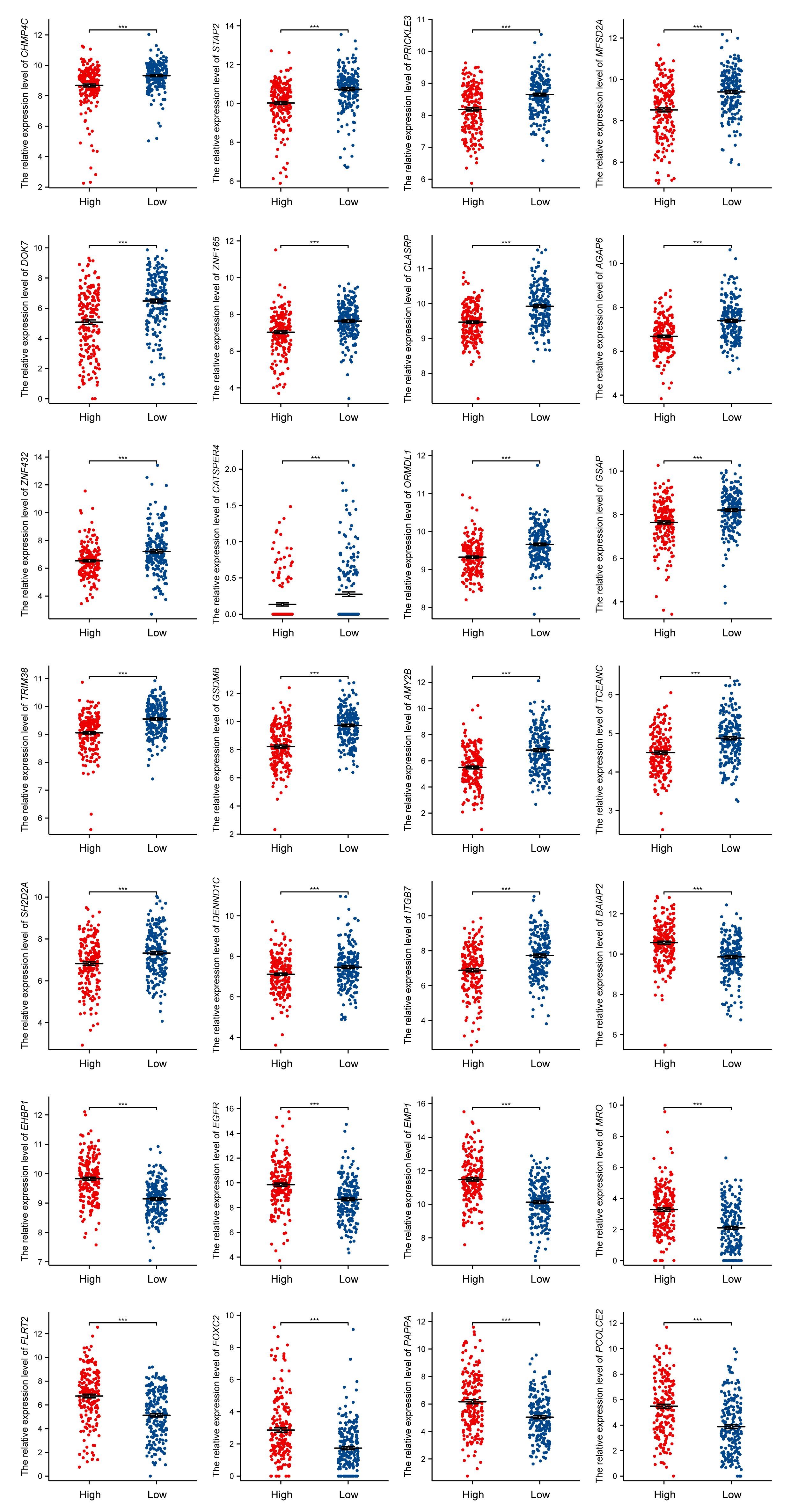

Supplement: Supplementary file 6 [file Image_5.tif]

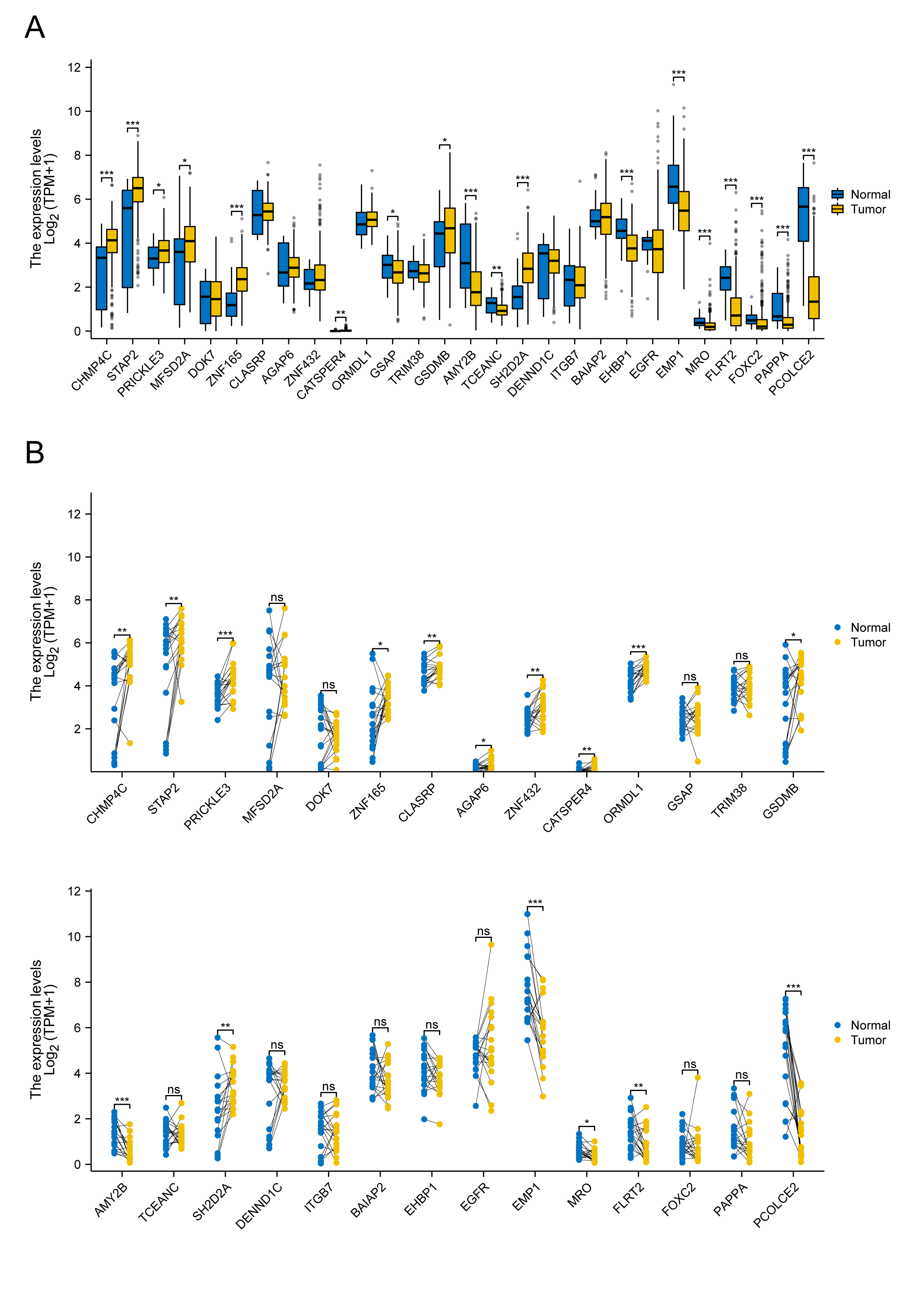

Supplement: Supplementary file 7 [file Image_6.tif]

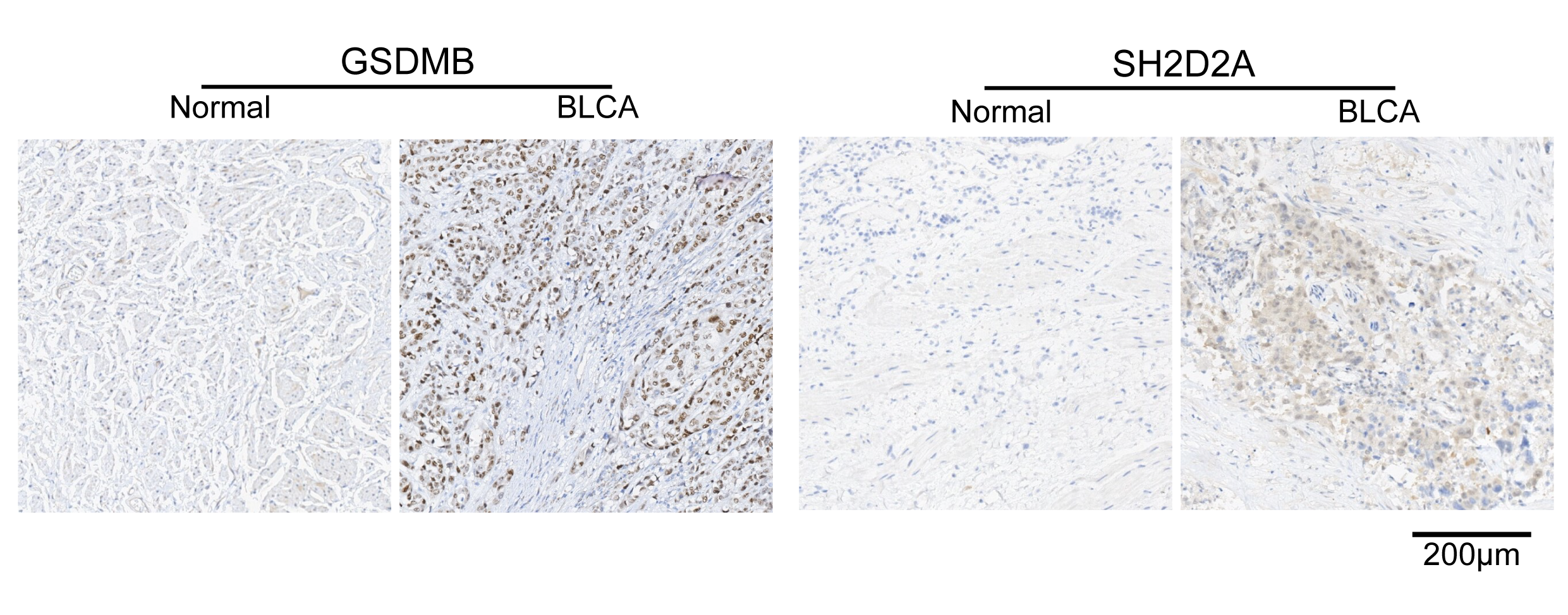

Supplement: Supplementary file 8 [file Image_7.tif]
