# Supplementary material for: Construction of T cell exhaustion model for predicting survival and immunotherapy effect of bladder cancer based on WGCNA
Source: Front Oncol. 2023 May 30;13:1196802. doi: 10.3389/fonc.2023.1196802 (PMC10266200; doi:10.3389/fonc.2023.1196802)
Supplement: Supplementary file 9 [file Table_1.docx]

Table S1. The clinicopathological characteristics of 23 patients with bladder cancer.

| Patient ID | Age (years) | Sex | Previous history of BLCA | Smoking history | drinking history | surgical procedure | Types of pathologies |
| --- | --- | --- | --- | --- | --- | --- | --- |
| #1 | 65 | Male | Yes | Yes | Yes | TURBT^1^ | sUC^3^ |
| #2 | 75 | Male | Yes | Yes | Yes | TURBT | IUC^4^ |
| #3 | 79 | Female | No | No | No | RC^2^ | IUC |
| #4 | 76 | Male | No | No | No | TURBT | MPUC^5^ |
| #5 | 58 | Male | Yes | Yes | No | RC | IUC |
| #6 | 71 | Male | No | Yes | Yes | TURBT | MPUC |
| #7 | 61 | Male | Yes | Yes | Yes | TURBT | IUC |
| #8 | 75 | Male | No | No | No | TURBT | IUC |
| #9 | 62 | Male | No | No | Yes | TURBT | MPUC |
| #10 | 69 | Male | No | Yes | Yes | TURBT | sUC |
| #11 | 71 | Male | No | Yes | No | TURBT | IUC |
| #12 | 53 | Male | No | No | Yes | TURBT | MPUC |
| #13 | 75 | Male | Yes | Yes | Yes | TURBT | IUC |
| #14 | 64 | Male | No | Yes | Yes | TURBT | MPUC |
| #15 | 74 | Female | No | No | No | TURBT | IUC |
| #16 | 52 | Male | No | No | No | RC | MPUC |
| #17 | 59 | Female | No | No | No | RC | IUC |
| #18 | 65 | Male | No | No | No | TURBT | MPUC |
| #19 | 65 | Male | No | No | No | TURBT | MPUC |
| #20 | 73 | Male | Yes | Yes | Yes | RC | sUC |
| #21 | 80 | Male | No | Yes | Yes | RC | IUC |
| #22 | 73 | Male | No | No | No | TURBT | IUC |
| #23 | 83 | Male | No | Yes | Yes | TURBT | IUC |

1 TURBT, Transuretheral resection of bladder tumor

2 RC, Radical cystectomy

3 sUC, situ urothelial carcinoma

4 IUC, Invasive urothelial carcinoma

5 MPUC, Micropapillary urothelial carcinoma
