# Supplementary material for: Construction of T cell exhaustion model for predicting survival and immunotherapy effect of bladder cancer based on WGCNA
Source: Front Oncol. 2023 May 30;13:1196802. doi: 10.3389/fonc.2023.1196802 (PMC10266200; doi:10.3389/fonc.2023.1196802)
Supplement: Supplementary file 10 [file Table_2.docx]

Table S2. The primers of twelve characteristic genes and GAPDH used in qPCR

| Gene Name | Forward Primer | Reverse Primer |
| --- | --- | --- |
| AMY2B | TGCATAGAAATGGCACATAGT | AAATTAACTCCATGCATCACC |
| ZNF165 | ACCAAGGCCCATTTTGATTCA | CTCTGAGACTCCCCTGATTCTT |
| EMP1 | GTGCTGGCTGTGCATTCTTG | CCGTGGTGATACTGCGTTCC |
| MRO | TAAAAAGCGTCACATGGCAATG | GCAGGTCGAGGACAATTTTCTT |
| EHBP1 | TGGTTGAGTGTACGAAGAAATGG | ACAACACCACGATAGGGATTTTT |
| FLRT2 | CGCTGCGACAGGAACTTTG | TGGAGGTAGAGTACGGTTACG |
| GSDMB | TGATTGCCGTTAGAAGCCTTG | TCCCGTTGAGTCTACATTATCCA |
| STAP2 | GTCCCCAAGCCTAAGGGTG | TGGAAGTCCCGATTGCTATTGTA |
| CHMP4C | TGGTCCGACTTCGGGAGAC | GCCAGGGCGATTTCTCTCTG |
| PCOLCE2 | TACTTGGAAAATCACAGTTCCCG | CGGCACAGGTTGTCACTCTC |
| SH2D2A | GACTTTCCCTGAGGACCGAAG | GCTTGCCCCTGTTTGATGATTG |
| PRICKLE3 | CTGGAACGCATCATGTGTCG | CCCAGGCATACTCCTCCGAT |
| GAPDH | GTCTCCTCTGACTTCAACAGCG | ACCACCCTGTTGCTGTAGCCAA |
