# Supplementary material for: Construction of T cell exhaustion model for predicting survival and immunotherapy effect of bladder cancer based on WGCNA
Source: Front Oncol. 2023 May 30;13:1196802. doi: 10.3389/fonc.2023.1196802 (PMC10266200; doi:10.3389/fonc.2023.1196802)
Supplement: Supplementary file 11 [file Table_3.docx]

Table S3. Antibody information used in IHC experiments.

| Antibodies | Host | Dilution Ratio | Danufacturer | Catalog number |
| --- | --- | --- | --- | --- |
| ZNF165 | Rabbit | IHC: 1:400 | Invitrogen | PA5-21546 |
| SH2D2A | Rabbit | IHC: 1:300 | Invitrogen | PA5-106742 |
| GSDMB | Rabbit | IHC: 1:250 | Invitrogen | PA5-62601 |
| PRICKLE3 | Rabbit | IHC: 1:200 | Invitrogen | PA5-98087 |
| CHMP4C | Rabbit | IHC: 1:200 | Invitrogen | PA5-54889 |
| STAP2 | Rabbit | IHC: 1:50 | Invitrogen | PA5-82014 |
| HRP | Goat Anti-Rabbit | IHC: 1:1000 | Abcam | ab6721 |
